# Biopolymer Lipid Hybrid Microcarrier for Transmembrane Inner Ear Delivery of Dexamethasone

**DOI:** 10.3390/gels8080483

**Published:** 2022-08-01

**Authors:** Maximilian George Dindelegan, Violeta Pașcalău, Maria Suciu, Bogdan Neamțu, Maria Perde-Schrepler, Cristina Maria Blebea, Alma Aurelia Maniu, Violeta Necula, Anca Dana Buzoianu, Miuța Filip, Alexandra Csapai, Cătălin Popa

**Affiliations:** 1 Department of Clinical Pharmacology, “Iuliu Hatieganu” University of Medicine and Pharmacy, 23 Gh. Marinescu Street, 400337 Cluj-Napoca, Romania; maximilian.dindelegan@gmail.com (M.G.D.); abuzoianu@umfcluj.ro (A.D.B.); 2 Department of Otorhinolaringology, “Iuliu Hatieganu” University of Medicine and Pharmacy, 4-6 Clinicilor Street, 400006 Cluj-Napoca, Romania; cristina_blebea@yahoo.com (C.M.B.); almacjro@yahoo.com (A.A.M.); neculav@yahoo.com (V.N.); 3 Department of Materials Science and Engineering, Technical University of Cluj-Napoca, 28 Memorandumului Street, 400114 Cluj-Napoca, Romania; bogdan.neamtu@stm.utcluj.ro (B.N.); alexandra.csapai@stm.utcluj.ro (A.C.); catalin.popa@stm.utcluj.ro (C.P.); 4 Electron Microscopy Center “C. Craciun”, Biology and Geology Faculty, Babes-Bolyai University, 5-7 Clinicilor Street, 400006 Cluj-Napoca, Romania; suciu.maria@ubbcluj.ro; 5 National Institute for Research and Development of Isotopic and Molecular Technologies, 67-103 Donath Street, 400293 Cluj-Napoca, Romania; 6 Institute of Oncology “Prof Dr. Ion Chiricuta”, 34-36 Republicii Street, 400015 Cluj-Napoca, Romania; pmariaida@yahoo.com; 7 “Raluca Ripan” Institute for Research in Chemistry, Babes-Bolyai University, 30 Fantanele Street, 400294 Cluj-Napoca, Romania; miuta.filip@ubbcluj.ro

**Keywords:** microparticles, Lipoid S 100, pectin, BSA, hydrogel

## Abstract

Dexamethasone is one of the most often used corticosteroid drugs for sensorineural hearing loss treatment, and is used either by intratympanic injection or through systemic delivery. In this study, a biopolymer lipid hybrid microcarrier was investigated for enhanced local drug delivery and sustained release at the round window membrane level of the middle ear for the treatment of sensorineural hearing loss (SNHL). Dexamethasone-loaded and dexamethasone-free microparticles were prepared using biopolymers (polysaccharide and protein, pectin and bovine serum albumin, respectively) combined with lipid components (phosphatidylcholine and Dimethyldioctadecylammonium bromide) in order to obtain a biopolymer–liposome hybrid system, with a complex structure combining to enhance performance in terms of physical and chemical stability. The structure of the microparticles was evaluated by FTIR, XRD, thermal analysis, optical microscopy, and scanning electron microscopy (SEM). The encapsulation efficiency determination and the in vitro Dexamethasone release study were performed using UV-Vis spectroscopy. The high value of encapsulation efficiency and the results of the release study indicated six days of sustained release, encouraging us to evaluate the in vitro cytotoxicity of Dexamethasone-loaded microparticles and their influence on the cytotoxicity induced by Cisplatin on auditory HEI-OC1 cells. The results show that the new particles are able to protect the inner ear sensory cells.

## 1. Introduction

Hearing loss represents a worldwide health issue. More than 1.5 billion people are affected by hearing loss, according to the “World Report on Hearing” published by the World Health Organization in 2021. A large number, approximately 430 million, suffer from moderate or worse hearing loss levels, which impact their quality of life and daily activities [1]. Sensorineural hearing loss can be genetically inherited or acquired. Acquired SNHL follows sensory inner ear cell destruction. The causes of inner ear cell destruction are represented by infectious pathogens (viral or bacterial), ototoxic drugs (aminoglycoside antibiotics, loop diuretics, platinum-based chemotherapeutics), traumatic causes, noise-induced hearing loss, and idiopathic hearing loss.

Sudden sensorineural hearing loss (SSNHL) is defined as SNHL of at least 30 dB over at least three contiguous frequencies occurring in a maximum of 72 h [2]. SSNHL has an incidence of 5–20 per 100,000 persons. In the majority of patients the etiology remains unknown, and thus their hearing loss is considered idiopathic [3]. About 98% of otolaryngologists in the U.S. state that they treat idiopathic SSNHL with oral steroids, and 8% reported using intratympanic administration of steroid drugs [4].

Platinum-based chemotherapeutics, such as Cisplatin, cause bilateral high-frequency sensorineural hearing loss [5]. Many patients who are undergoing treatment with cisplatin chemotherapy experience hearing loss, with studies suggesting that 40–80% are affected and experience permanent hearing loss [6]. Cisplatin induces hearing loss through an imbalance of the antioxidant defense system; increasing the reactive oxygen species increase the early immediate release and synthesis of pro-inflammatory cytokines, which induces apoptosis in inner ear cells [7]. Experimental studies on mice have shown that cisplatin-induced ototoxicity can be avoided by daily intratympanic dexamethasone injection for all sound frequencies except 32 kHz, where little protection was observed [8].

The most widely accepted and used treatment for acquired SNHL is represented by corticotherapy [9]. Corticosteroids can be administered through systemic delivery or local delivery. Systemic delivery of steroid drugs into the inner ear is restricted because of the presence of the blood–labyrinth barrier, which isolates the inner ear from the bloodstream [10], and can lead to important systemic side effects because of the high doses needed to reach therapeutic concentration [11]. The other option used in clinical practice for reaching therapeutic drug concentrations in the inner ear is represented by intratympanic delivery of substances. Injection of a drug through the tympanic membrane into the middle ear allows it to diffuse through the round window membrane and oval window membrane into the inner ear, effectively bypassing the blood–labyrinth barrier and avoiding the systemic side effects of the molecules [12]. The main disadvantages of transtympanic delivery are represented by clearing of the substance through the eustachian tube, resorption through the middle ear mucosa, the risk of otitis media, and persistent perforation of the tympanic membrane [13]. The clearance of the substances from the middle ear means that the intratympanic injection must be repeated multiple times throughout the treatment schedule. To counter clearing of the drugs from the level of the middle ear, various prolonged drug delivery systems have been proposed [14,15]. Developing a stable and safe prolonged drug delivery platform at the level of the inner ear would lead to a paradigm shift for corticosteroid treatment in clinical practice, effectively removing the need for repeated injections through the tympanic membrane into the middle ear.

Dexamethasone (Dexa) is a corticosteroid drug that has been used in the treatment of sudden sensorineural hearing loss both by systemic administration and by intratympanic injection in clinical settings [16,17]. Compared to prednisone and prednisolone, it has minimal mineralocorticoid effect and a longer duration of action [18,19].

Developing microcarriers capable of prolonged local delivery of corticosteroid drugs into the middle ear and from there into the inner ear could represent a viable solution for the treatment of patients suffering from treatable acquired SNHL (ototoxic hearing loss, SSNHL).

Our approach is based on a liposomal-type microcarrier with enhanced structure for Dexa loading, delivery, and sustained release, and with major components that are natural. Liposomes are self-assembled phospholipid vesicles composed of an aqueous compartment surrounded by one or more phospholipid bilayer membranes. Although liposomes offer several advantages over other carrier systems, such as the ability to carry both hydrophobic and hydrophilic compounds, high encapsulation efficiency, biocompatibility, and transport ability through cell membranes, they are thermodynamically unstable systems prone to aggregation, fusion, degradation, or hydrolyzation, resulting in leakage of the entrapped compounds. The main limitations of liposome application in drug delivery systems are their physical and chemical instability [20,21] and their retention time after both systemic and local application [22]. Hydrophobic interactions are responsible for their physical instability, while degradative lipid oxidation determines their chemical instability. Our intent was to improve the physical stability of liposomes by introducing both a polysaccharide and a protein in the liposome structure to increase repulsion forces through both steric and electrostatic interactions.

The use of biopolymers such as polysaccharides and proteins has led to a second generation of liposomes, namely, biopolymer lipid hybrid (PLH) systems, which have improved in vitro and in vivo performance based on the cumulative benefits of the polymer and lipid together [23]. From the many types of PLH, we have chosen ones with a modified surface in order to improve the system’s structural stability in the case of changes in shape, size, surface charge, or lipid chain ordering [24]. We synthesized a polysaccharide protein–lipid hybrid system which can offer advantages of biodegradability, biocompatibility, and lower toxicity. Lipids, one of the major human nutrients, are biocompatible and biodegradable. The safety and diversity of lipids have attracted interest to their applications in drug delivery.

The lipid phosphatidylcholine contained soy lecithin (Lipoid S100) (L), and Dimethyldioctadecylammonium bromide (DDAB) was used for the synthesis of the surface coated liposomes. Phosphatidylcholine is one of the most widely used lipids in liposome preparation, and DDAB is another lipid component which contributes to increasing the electrostatic interactions with the other components of the system. 

As a polysaccharide, we chose pectin (P), an anionic polysaccharide composed of galacturonic acid units linked by α-1,4 bonds. It has been demonstrated that interaction of P with egg lecithin liposomes can regulate the freedom of lipid molecules and enhance their rigidity and mechanical strength [25]. Natural P is characterized by different degrees of carboxylic group methoxylation, being conventionally designated as low (LMP) and high methoxylated (HMP) P, respectively. Studies have compared the performance of the two kinds of P on the stability of liposomes. The reported results highlight higher stability for LMP-coated liposomes, which is provided by the higher amount of carboxylic groups; these make the surface more negative, increasing electrostatic repulsion between particles and allowing LMP to better bind to the liposome surface through hydrogen bonding. In addition, LMP has increased availability for participation in crosslinking reactions with metal cations, forming stable hydrogel networks on the coated liposome surfaces [26,27]. P is a water-soluble and edible polysaccharide, and in addition to its emulsifying, suspending, and hydrogel-forming properties it acts as a mucoadhesive agent as well, sustaining drug release for rectal and nasal drug delivery [28,29].

The choice of Bovine serum albumin (BSA) as a protein candidate for building the microcarrier was based on its intrinsic biodegradability, biocompatibility, and lack of toxicity and immunogenicity [30,31], being intensively used as a protein model for transporting different hydrophobic macromolecules or small drug molecules to target sites [32,33]. BSA is an ampholyte with a pH-dependent charge, providing reversible sites to bind and release drugs [34,35].

Our approach is based on a modified dispersion technique for liposome preparation, which has the advantage of being the most well suited for passive loading of hydrophobic drugs with high encapsulation efficiency [36,37]. We studied and selected the optimal parameters for processing free (Lmp) and Dexa-loaded (Lmp/Dexa) Lipid/pectin/BSA microparticulate systems, considering the concentrations and sequence of adding the components, stirring speed, temperature, incubation time, separation, and storage conditions, as well as samples preparation for physical chemical characterization. On the other hand, the choice of the Dexa encapsulation pathway in the microparticle synthesis stage has the advantage of allowing a high degree of encapsulation and represents a way of ensuring the protection of the drug while not subjecting it to several stages of processing, which would affect its structure. The use of both polysaccharides and proteins to improve the system’s stability and sustain drug delivery by increasing repulsion forces through steric and electrostatic interactions and by building a protective shell against degradative lipid oxidation is the novelty of our study. The results achieved in physical chemical characterization of the obtained microparticles confirm the complex structure as well as the stability of the developed system. The in vitro release process of Dexa and in vitro cytotoxicity on the House Ear Institute-Organ of Corti 1 (HEI-OC1) cell line and the spontaneously immortalized human keratinocyte cell line HaCaT, along with the influence on the cytotoxicity induced by Cisplatin in HEI-OC1 cells of the new system, were all assessed.

The complex architecture of Lmp/Dexa as a hybrid biopolymer lipid system offers both stability and multifunctionality, making the microcarrier intelligent and suitable for specific local applications of Dexa. To the best of our knowledge, this is the first report to propose this approach.

## 2. Results and Discussion

### 2.1. Encapsulation Efficiency of Dexa (%) and Dexa Loading Efficiency (%)

The hydrophobicity of Dexa was the main reason for choosing to build the above biopolymer lipid hybrid architecture. Dexa loading in Lmp was achieved in the same stage as microparticle processing by dispersing the ethanolic solution in which Dexa and the two lipids were dissolved into the aqueous solution containing the hydrophilic components (P–Calcium crosslinked hydrogel and BSA). The Dexa-loaded Lmp is denoted Lmp/Dexa. Table 1 shows the mean values of E_ef_ (%) and Dexa Load_ef_ (%) (g/100g), calculated based on Equation (1) for all three variants of microparticles (Lmp/Dexa) achieved, taking into account three sample preparations. It should be noted that the efficiency of the encapsulation is influenced by the Dexa concentration used in the preparation, which allows variants of microparticles with Dexa content suitable for specific applications. As the experimental data show, one way to increase the loading efficiency is to use a higher concentration of Dexa in the preparation phase of the microparticles; the process parameters can be optimized as well. A possible option to increase the loading efficiency could be the use of a variety of water-soluble Dexa, such as Disodium Dexa Phosphate, which would allow much higher Dexa concentrations. This will be one of our concerns in the future.

The high value of E_ef_ (%) should be due to the hydroxy groups of Dexa involved in hydrogen bonds with the hydroxy groups of P and BSA, as well as to increased electrostatic interactions with Phosphatidylcholine (Lipoid S100) and DDAB. In this respect, Dexa loading in Lmp was favored in both the biopolymer coating and in the core of the hybrid system. The first variant was chosen, with the ratio 2:10:1, and throughout the paper we refer to it under the name Lmp/Dexa.

### 2.2. Lmp and Lmp/Dexa Structure Characterization

#### 2.2.1. Fourier-Transform Infrared Spectroscopy

**Figure 1:**
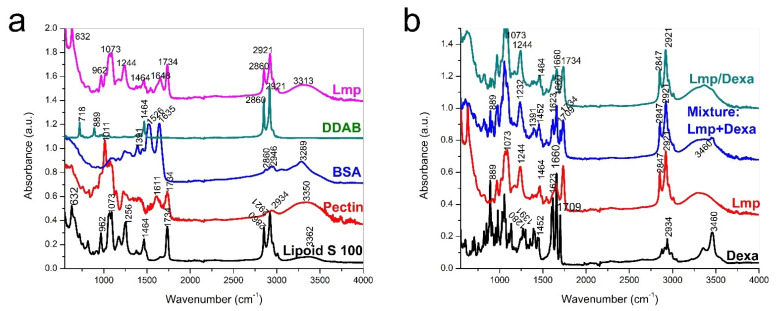
Overlapped FTIR spectra of: Lmp and the precursors (Lipoid S 100, P, BSA and DDAB) used for Lmp synthesis (**a**); pure Dexa, Lmp, Physical mixture of Lmp + Dexa and Lmp/Dexa (**b**).

Fourier-transform infrared (FTIR) spectra of the precursors (Lipoid S 100, P, BSA, DDAB) as well as the free (Lmp) and Dexa-loaded (Lmp/Dexa) formulations achieved in the experimental section are shown in Figure 1. Interpretation of the spectra can provide information about the chemical structure of the synthesized microstructures and the Dexa encapsulation process. The FTIR spectra may highlight, where applicable, the new bonds formed between Dexa and Lmp and the disappearance of bonds from the precursors. As can be seen in Figure 1a, the Lmp spectrum shows characteristic bands of P, Phosphatidyl choline, BSA, and DDAB. The vibration of the cyclic etheric band at 1011 cm^−1^ from P overlaps with those of alkyl ether from Phosphatidyl choline at 1073 cm^−1^, the vibration bands of OH and C–H bonds, which appear at 3313, 2860, and 2921 cm^−1^, respectively, and specific bands of P and Phosphatidyl choline such as the asymmetric and symmetric stretch of COO– at 1611 cm^−1^ and 1464 cm^−1^ and the stretch vibration due to the C=O carboxylic acid from P and C=O of ketone from Lipoid S100 at 1734 cm^−1^.

The two amide-specific bands of BSA (particularly the in-plane bend vibration band at 1528 cm^−1^ and the stretching vibration band of the C=O bond at 1635 cm^−1^) can both be observed in the BSA, and are superimposed with those of the carboxylate bands into a broad band in the Lmp spectrum, the a result of low BSA content of in the structure. The FTIR spectrum of pure Dexa (Figure 1b) shows multiple bands due to the vibrations of the chemical structure with many functional groups in the molecule: –C–F stretch—1432 cm^−1^; ketone C=O stretch—1709 cm^−1^; C–H stretch—2934 cm^−1^; HO– stretch—3460 cm^−1^. The FTIR spectrum of Lmp/Dexa, shows bands provided by both Lmp and Dexa, most of them being superimposed. No major differences in the FTIR spectra of physical mixture Lmp + Dexa and Lmp/Dexa were observed, thus, the FTIR tests confirmed that there are no new bonds created between pure Dexa and the functional groups of Lmp.

#### 2.2.2. X-ray Diffraction

**Figure 2:**
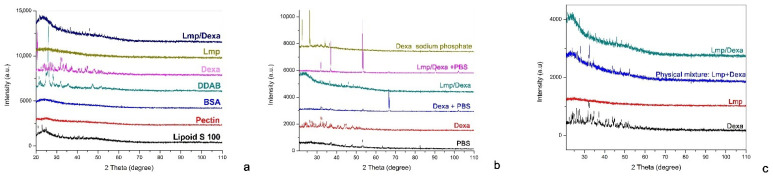
X-ray diffraction patterns of: Lmp and Lmp/Dexa and the precursors (Lipoid S 100, P, BSA and DDAB, Dexa) used for Lmp and Lmp/Dexa synthesis (**a**); dried PBS, Dexa, Dexa recovered from suspension in PBS, Lmp/Dexa, and Lmp/Dexa recovered from suspension in PBS, and Dexa sodium phosphate (**b**); pure Dexa, Lmp and the physical mixture of Lmp+Dexa, and Lmp/Dexa (**c**).

The XRD patterns of Lmp and Lmp/Dexa samples are shown in Figure 2 alongside those of the precursors used for their synthesis, the samples of Dexa, Lmp, and Lmp/Dexa, respectively, treated with PBS, and the Lmp + Dexa physical mixture. As can be seen in Figure 2a, Lmp is in amorphous state, with the diffraction pattern showing only a characteristic halo, although some of the precursors are in a crystalline state. In the same figure, the X-ray pattern of the pure Dexa reveals a crystalline state. The XRD pattern of Lmp/Dexa samples indicates the presence of two phases, one amorphous and the other crystalline. The crystalline phase can be assigned to Dexa because the diffraction peaks of Lmp/Dexa appeared at the same positions as in the case of the pure Dexa. However, certain intensities are not identical, indicating a preferential orientation of the recrystallized Dexa crystals due to spatial constraints, as reported by other authors [38,39]. The presence of Dexa in the Lmp/Dexa samples confirms the success of the loading process. In order to study the release of Dexa from Lmp/Dexa in PBS medium, the diffraction patterns of pure Dexa and Lmp/Dexa samples recovered from their suspensions in PBS were examined and compared with those of dried PBS, pure Dexa, and Dexamethasone sodium phosphate. The results are shown in Figure 2b. In the XRD pattern of the Dexa + PBS sample a new maximum can be seen, indicating that Dexa has undergone a transformation in its crystalline structure. As a result, the newly formed crystalline species, which could be a phosphate ester of Dexa, absorbs UV-Vis radiation corresponding to a wavelength other than that of pure Dexa, and measurement of the concentration of the new species using the calibration curve recorded for pure Dexa is no longer possible. These results confirm the experimental observations, and constitute an argument in favor of the decision to avoid conducting the study of release in PBS and instead conduct it in water containing a minimum amount of ethanol. Figure 2c compares the diffraction patterns of the Lmp + Dexa physical mixture with the Lmp/Dexa synthesized hybrid system. The two diffractograms are similar, leading to the conclusion that during the encapsulation process Dexa does not undergo polymorphic transformation.

#### 2.2.3. Thermal Analysis

**Figure 3:**
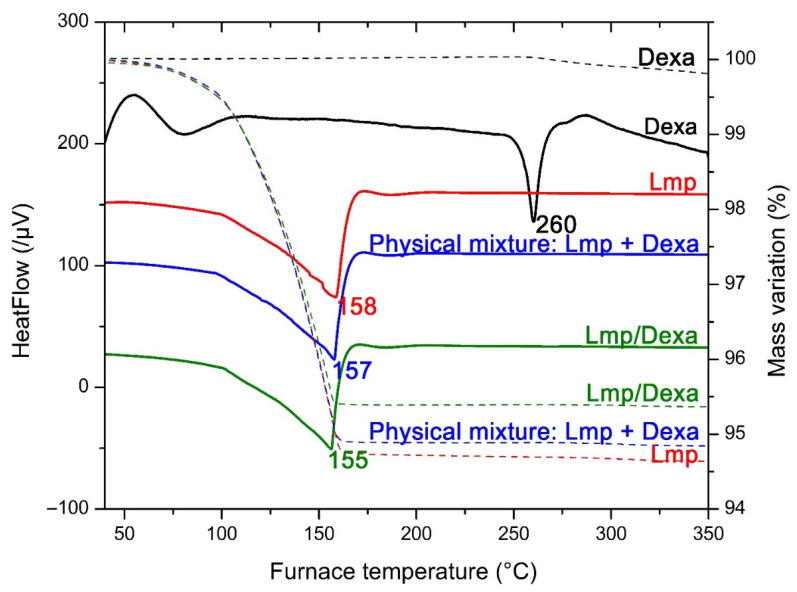
Overlapped DSC and TG thermograms of pure Dexa, Lmp, the physical mixture of Lmp+Dexa, and Lmp/Dexa.

The thermograms of pure Dexa, the free Lmp and Dexa encapsulated system, and the physical mixture between Lmp and Dexa are shown in Figure 3. The interaction between Dexa and the lipidic components of Lmp/Dexa was demonstrated by the decrease of the melting temperature (the endothermic peak), as can be seen in Figure 3, from 158 °C for Lmp to 155 °C for Lmp/Dexa and to 157 °C for the physical mixture between Lmp and Dexa. The Dexa molecules seem to be dissolved in the melted Lmp matrix, justifying the absence of the endothermic peak from 260 °C in the DSC thermograms of Lmp/Dexa and the physical mixture of Lmp + Dexa.

#### 2.2.4. Optical Microscopy

**Figure 4:**
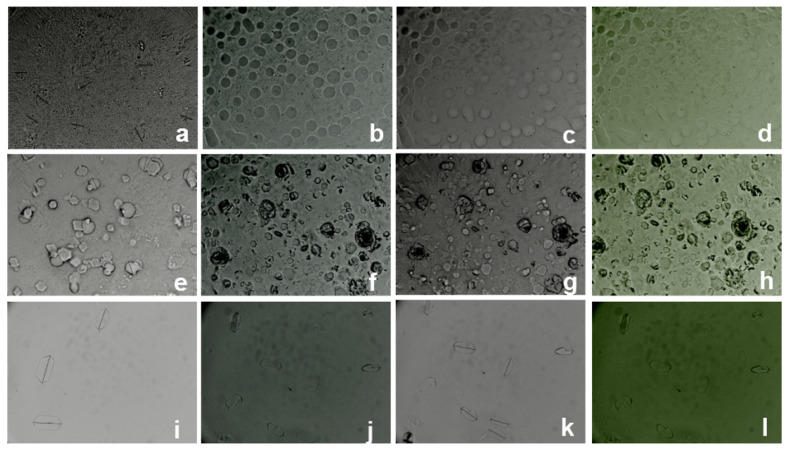
Optical images of: Lmp without Fluorescein (**a**) and Lmp with Fluorescein Blue filter (**b**), Bright field (**c**), and Green filter (**d**) (40×); Lmp/Dexa without Fluorescein (**e**) and Lmp/Dexa with Fluorescein Blue filter (**f**), Bright field (**g**), and Green filter (**h**) (40×); Free Dexa crystals recovered from the supernatant resulting from Lmp/Dexa without Fluorescein (**i**) and Lmp/Dexa with Fluorescein preparation Blue filter (**j**), Bright field (**k**), and Green filter (**l**) (40×).

Optical microscopy was used to observe the shape and size of both Lmp and Lmp/Dexa. The optical microscopy images (Figure 4) of the samples prepared without Fluorescein for (a) Lmp and (e) Lmp/Dexa and with Fluorescein for (b–d) Lmp and (f–h) Lmp/Dexa show particles of spherical shape, around 25 µm size in average, and with or without Dexa hexagonal crystals on the surface. Moreover, the free Dexa crystals (35 µm length) recovered from the supernatant resulting from Lmp/Dexa without Fluorescein (i) and Lmp/Dexa with Fluorescein preparation (j–l) are shown. Considering the fact that P makes up the majority of the shell that surrounds the lipid part of the microsystem, the charge is expected to be negative. Images of fluorescent samples suggest the presence of pectin hydrogel, capable of fixing fluorescein in the microparticle shell. This information is relevant for the stability profile of the microparticles as well as for their swelling behavior in aqueous environments. As can be seen, free Dexa crystals do not change their appearance when preparing microparticles with fluorescein.

#### 2.2.5. SEM

**Figure 5:**
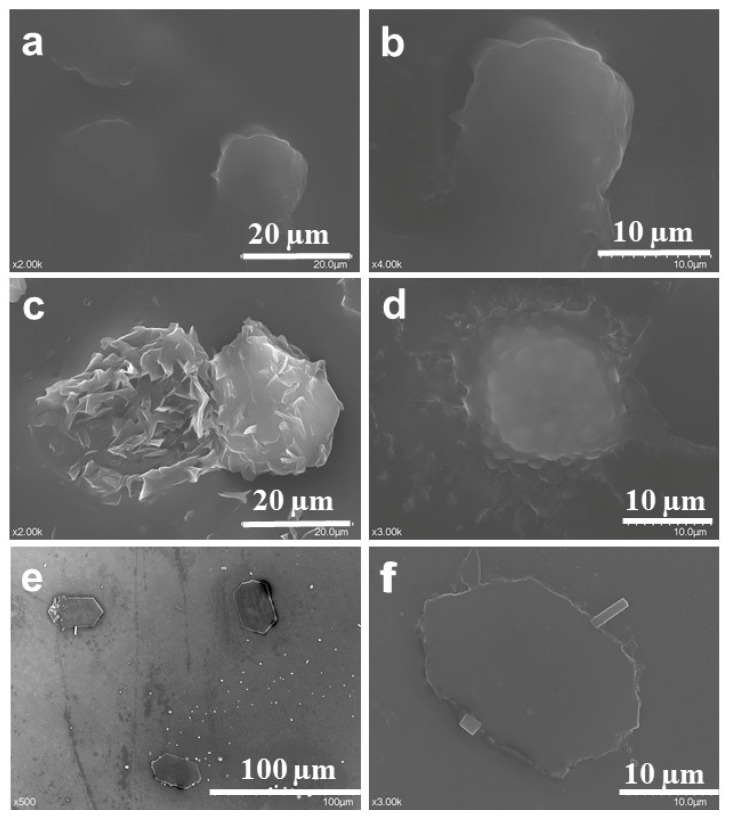
SEM microimages of Lmp (**a**,**b**), Lmp/Dexa (**c**,**d**), and Dexa crystals (**e**,**f**) from the supernatant separated after Lmp/Dexa sedimentation.2.3. Lmp/Dexa Charateristics.

The SEM micrographs (Figure 5) provide information about the shape and size of the examined particles as well as the presence of drug crystals on the surface. The SEM images highlight core-shell spherical microparticles around 20 µm in diameter. The shell seems to contain drug crystals.

### 2.3. Lmp/Dexa Charateristics

#### 2.3.1. In Vitro Release Study

**Figure 6:**
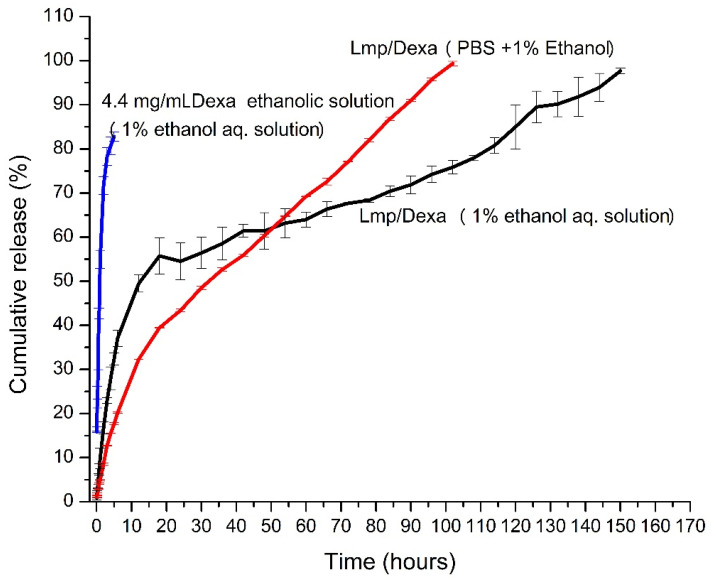
Cumulative release (%) versus time of Dexa released from Lmp/Dexa in 1% ethanol aqueous solution and in PBS containing 1% ethanol, and from Dexa ethanolic solution in 1% ethanol aqueous solution.

The release study of Dexa from Lmp/Dexa was performed in both water and PBS pH 7.4, each containing a small amount of ethanol (1%), which has the role of dissolving the released Dexa molecules. The supply of ethanol required for the continuous dissolution of Dexa molecules was ensured by replacing the volumes of samples removed from the release medium with identical volumes of fresh medium containing ethanol. Based on the Dexa concentrations determined from the collected samples from the release medium at different time intervals, the cumulative amounts of Dexa released were calculated, and taking into account the Dexa content of the Lmp/Dexa sample subjected to release, the cumulative release (%) values were calculated and plotted against time (Figure 6). The release profile of Dexa from pure Dexa solution of the same Dexa concentration as in the microparticles was performed, and is included in Figure 6. As expected, Dexa release from free Dexa solution has a different release profile than Lmp/Dexa release, being released almost completely in 2 h. As indicated in the release profile, Dexa is released from Lmp/Dexa continuously for six days, with a burst effect in the first 20 h in water. The release process seems to be favoured in PBS, with a decreased burst effect. After five days we observed the appearance of particles in suspension in the release medium, both in water and in PBS, which indicates a process of degradation of the studied system. This process could be caused by the multiple openings of the system in order to remove the samples taken from the release medium, and would of course be favored by the aqueous environment and the temperature of 37 °C. The release of Dexa into water is favored by the presence of the pectin hydrogel crosslinked with Ca^2+^ ions in the particle shell, as their network hydrates, swells, and dilates the pores, thus allowing small Dexa molecules to diffuse into the release medium. Although the ethanol molecules have the opposite effect of dehydrating the hydrogel network by competing for the water molecules with which they form stable hydrogen bonds, the very low ethanol content in the release medium can only slightly counterbalance the hydration process, swelling, and enlarging of the network pores. The presence of ethanol can lead to a slight prolongation of the release process. Comparing the process of release in diluted ethanol solution with that of release in pure water is not possible, because the released Dexa molecules do not dissolve in water, and thus cannot be quantified by the above-mentioned method. The results of the study certainly indicate a prolonged release of Dexa from Lmp/Dexa for six days, which recommends the use of the system studied in the in vitro and in vivo experiments for the development of a suitable and precisely controlled delivery system for inner ear therapy.

The time length of sustained drug release can vary depending on the nature of the carrier particles, their size, the degree of drug loading, and the nature and intensity of the physical or chemical interactions in which the active compound is involved. In general, this range is shorter for nanoparticles and longer for microparticles used as carriers [40,41,42]. There are exceptions if the drug is chemically bonded to the functional groups belonging to the carrier matrix or to a high density of cationic charges that form strong electrostatic interactions with the anionic charges of the drug.

The Dexa release profile from Lmp/Dexa indicates an extended release of up to six days with a burst effect at the beginning of the time interval, which is in agreement with other reported results [43]. This may be due to the high value of loading, as well as to the interactions between Dexa and BSA, which is an ampholyte with a pH-dependent charge, presenting reversible sites to bind and release drugs as well as P and the lipid components.

Further studies on improving of the protective coating of the microparticles through the formation of polyelectrolytic complexes between anionic and cationic polysaccharides used in the shell building process are able to provide extra stability and sustain a longer release time.

#### 2.3.2. Cytotoxicity Assay of Lmp and Lmp/Dexa

##### Cell Viability Assays

**Figure 7:**
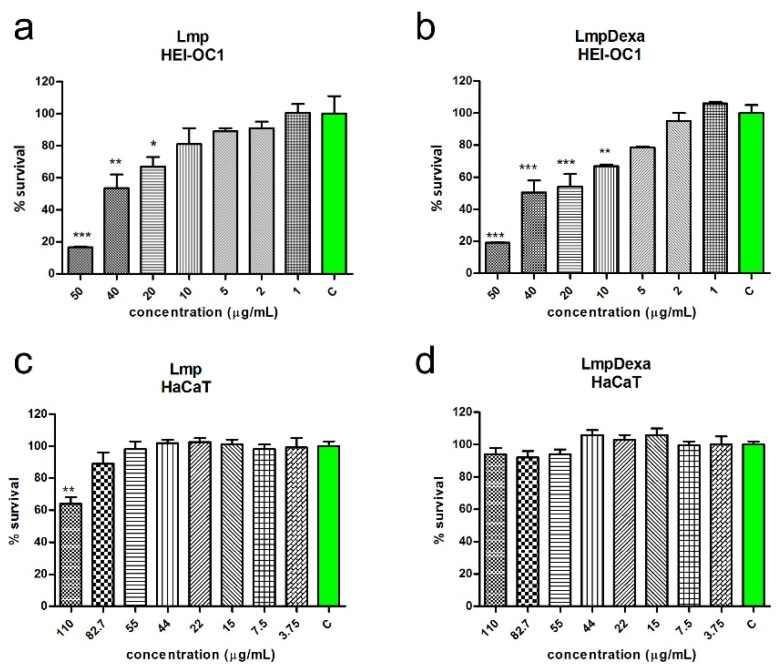
Concentration-dependent reduction of viability in HEI-OC1 and HaCaT cells after treatment with Lmp (**a**,**c**) and Lmp/Dexa (**b**,**d**) (*** *p* < 0.001; ** *p* < 0.01; * *p* < 0.5; one-way ANOVA, Dunnett’s multiple comparisons test).

The results regarding the cell viability assays after treatment of HEI-OC1 cells and HaCaT cells with Lmp and Lmp/Dexa, respectively, are illustrated in the Figure 7. Lmp and Lmp/Dexa both reduced the percentage of viable HEI-OC1 cells in a concentration-dependent manner. Concentrations below 10 µg/mL showed no significant reduction in viability compared to the control cells.

When tested on HaCaT keratinocytes, none of the Lmp samples (with or without Dexa) showed significant toxicity except when the highest concentrations were applied (110 and 82.7 µg/mL).

Our tests showed significant differences between the effects on the viability of the two cell lines used.

These differences in behavior may be explained by different physiological functions and differences in cellular uptake [44]. Studying 23 types of NPs in ten different cell lines, Kroll et al. reported differences in the sensitivity of the cell lines [45].

Another difficulty in determining whether a drug is cytotoxic lies in the sensitivity of the in vitro system. In vivo, in addition to the metabolism of a drug there are many other factors which determine its circulatory levels, such as binding to serum proteins, renal excretion, etc., and one cannot predict the final concentration that a cell will meet. In vitro, if the concentrations of most drugs under investigation is raised there is a level at which most, if not all, become cytotoxic [46].

Our results demonstrate that the sensitivity towards nanomaterial exposure is not only cell-type-specific, but also depends on the particle type used [41].

##### Protective Effect of Dexamethasone and Dexamethasone-Loaded Lmp against Cisplatin

Furthermore, we analyzed whether the new particles could protect inner ear cells against the cytotoxicity of Cisplatin. As reported in several previous studies, the treatment of HEI-OC1 cells with cisplatin induced a significant dose-dependent decrease in viability [47]. The IC50 of the concentration, reducing cell viability by 50%, was 65.79 µM.

**Figure 8:**
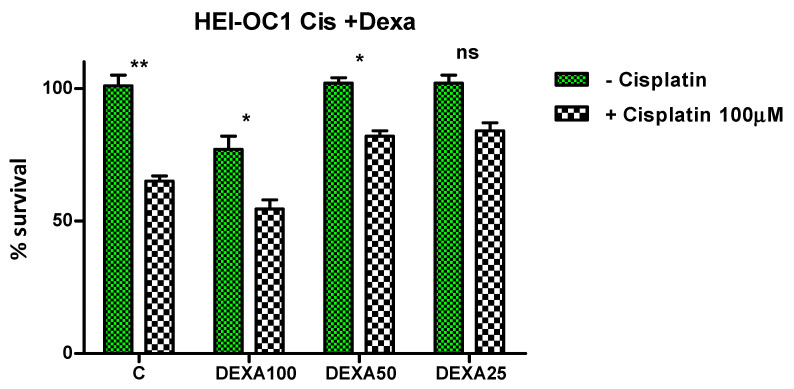
Protection of HEI-OC1 cells by pretreatment with Dexamethasone. Two-way ANOVA, Bonferroni post-tests. (** *p* < 0.01; * *p* < 0.5).

In our experiments, we used a concentration of 100 µM Cis, which is above the IC50 concentration, in order to induce cell toxicity. We then treated the HEI-OC1 cells with Dexamethasone in different concentrations, followed by 100 µM Cis. We obtained a reduction in toxicity for Dexamethasone at the concentrations of 50 and 25 µg/mL, respectively (Figure 8).

Dexamethasone is a synthetic fluorinated corticosteroid with multiple effects on practically every auditory cell, and it is frequently used in clinics to protect the auditory organ against inflammatory responses induced by noise, drugs, and other ototoxic stimuli. As with other drugs investigated in a study by Kalinec et al. [48], dexamethasone induced a decrease in cell viability without any significant change in cell death or caspase activation.

**Figure 9:**
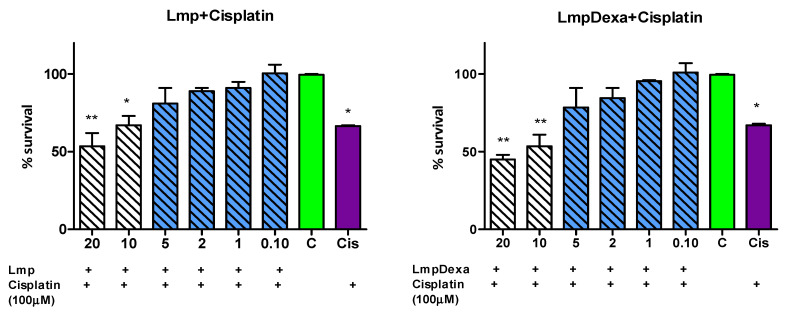
Lmp and Lmp/Dexa effect on cisplatin treated HEI-OC1 cells. (One-way ANOVA, Dunnett post-test). (** *p* < 0.01; * *p* < 0.5).

When we treated HEI-OC1 cells with Lmp in concentrations that were non-toxic (below 20 µg/mL) followed by 100 µM Cisplatin at concentrations of 5, 2, 1, and 0.1 µg/mL, no significant decreases in cell viability were observed compared to the non-treated cells for both types of microcapsules. (one-way ANOVA, Dunnett post-test) (Figure 9).

Our results show that the new particles, Lmp and Lmp/Dexa, are able to protect the inner ear neurosensorial cells from cisplatin toxicity in an in vitro model represented by the HEI-OC1 cell line. Dexa, as shown in our study, provides protection from cisplatin-induced toxicity in vitro. Particles that can continuously release Dexa in a controlled and prolonged manner could deliver a more efficient treatment option for different types of sensorineural hearing loss. These results point to a promising role for the newly described particles in hearing loss treatment. Further functional and anatomical in vivo studies are needed in order to test the otoprotection provided by Lmp/Dexa before the drug-delivery system can be considered a viable solution in clinical practice.

## 3. Conclusions

In our study, the main objective of developing a biopolymer lipid hybrid microcarrier for enhanced local Dexamethasone delivery and sustained release for the treatment of SNHL was accomplished. Dexamethasone-loaded and Dexamethasone-free microparticles were prepared using biopolymers (polysaccharide–protein and pectin–bovine serum albumin, respectively) combined with the lipid components (Lipoid S 100 and Dimethyldioctadecylammonium bromide) into a biopolymer–liposome hybrid system with a complex structure that combines for enhanced performance in terms of both physical and chemical stability. Structural characterization was achieved and in vitro study of Dexa release and cytotoxicity on the HEI-OC1 and HaCaT cell lines was carried out. The FTIR tests confirmed no new bonds are created between pure Dexa and the functional groups of Lmp. In addition, XRD patterns highlight that during the encapsulation process Dexa does not undergo polymorphic transformation. Images of fluorescent samples suggest the presence of pectin hydrogel in the shell of the microparticles, responsible for the stability profile of the microparticles as well as for their swelling behavior in aqueous environments. The results obtained in synthesis and characterization of the new microparticles, Lmp and Lmp/Dexa, including sustained release for up to six days and the lack of cytotoxicity to the HEI-OC1 cell line in the in vitro studies, are encouraging for the initiation of further work regarding the use of Lmp/Dexa in an in vivo functional hearing study. The sustained release of Dexa from Lmp/Dexa makes Lmp/Dexa more eligible for local delivery in the middle ear than a pure Dexa solution. Furthermore, the in vitro studies performed here will be followed by prospective in vivo studies on experimental animals in order to demonstrate the suitability of this microcarrier alone or when included in a hydrogel network as a potential vehicle for Dexa in the treatment of sensorineural hearing loss.

## 4. Materials and Methods

### 4.1. Materials

Phosphatidylcholine from Soybean Lipoid S 100 was kindly provided by Lipoid Gmbh (Ludwigshafen, Germany) Lipoid GmbH, Germany. Dexamethasone, Dexamethasone sodium phosphate, Dimethyldioctadecildiammonium bromide ≥ 98%, bovine serum albumin (BSA), albumin fraction V for biochemistry, Calcium chloride, and pectin from citrus peel ≥ 74% galacturonic acid were all purchased from Merck (Darmstadt, Germany). Sterile filtered and endotoxin tested Dulbecco’s phosphate buffered saline (PBS) and all the reagents used in biological studies were purchased from Sigma Life Science/Merck (Darmstadt, Germany)SIGMA Life Science. Ethanol 96% was purchased from S.C. Nordic Chemicals SRL, Cluj-Napoca, Romania.

### 4.2. Methods

#### 4.2.1. Preparation of Lipid/Pectin/BSA Microparticles (Lmp)

For Lmp preparation, we used the modified solvent dispersion technique [37,42,49,50]. A 0.2% P solution obtained by P dissolution in distilled water under vigorous magnetic stirring (1000 rpm) with heating up to 90 °C, filtrated on yellow filter paper and cooled at room temperature, was mixed with 0.1% BSA water solution at a ratio of 2:1 (*w/w*) until it homogenized. The mixture was incubated at 30 °C under slow magnetic stirring (200 rpm). A 1% CaCl_2_ (10% CaCl_2_ relative to the solid P) aqueous solution was added under continuous stirring. An ethanolic solution previously made by successively adding Lipoid S 100 and DDAB at a ratio of 1:10 (*w*/*w*) was injected with a 2 mL syringe. Incubation was maintained at 30 °C without stirring for 15 min. P and Lipoid S100 were in a 1:5 ratio (*w*/*w*). The obtained suspension containing Lmp was stored at 4 °C for minimum of 24 h before use. After removal of the supernatant, the sediment containing Lmp was stored at 4 °C before use.

#### 4.2.2. Preparation of Dexa-Loaded Lipid/Pectin/BSA Microparticles (Lmp/Dexa)

For Dexa-loaded microparticles (Lmp/Dexa), the same procedure described above was used, with Dexa being introduced together with the Lipoid S 100 and DDAB ethanolic solution. Three different concentrations of Dexa were used in order to determine the influence of its concentration on the microparticles’ encapsulation efficiency. In this regard, a multicomponent ethanolic solution was prepared by successively adding the components, after each complete dissolution, in the following order: Dexa, Lipoid S100, and DDAB in Ethanol 96%, under agitation and at room temperature, in a ratio of 2:10:1; 3:10:1 and 4:10:1 Dexa:Lipoid and S100:DDAB (*w*/*w*). Dexa and P were used 1:1 (*w*/*w*). Similarly, the addition of the ethanolic solution in the mixture of P and BSA solutions was carried out by incubation at 30 °C and 200 rpm, drop-wise, using a 2 mL syringe while maintaining incubation and without stirring after complete addition. The suspension containing Lmp/Dexa was kept at 4 °C for a minimum of 24 h for deposition. After removal of the supernatant, the sediment containing Lmp/Dexa was stored at 4 °C before use. The supernatants collected from each of the three prepared Lmp/Dexa variants were used to determine the free Dexa concentration, which was used for the further calculation of its encapsulation efficiency.

#### 4.2.3. Fluorescent Lmp and Lmp/Dexa Samples Preparation

In order to obtain information about the structure of the new developed hybrid biopolymer lipid microparticles, fluorescent microparticles were prepared using water-soluble Disodium Fluoresceinum. In this way, it is be possible to observe the position of the pectin hydrogel layer via fluorescence optical microscopy images, as it becomes fluorescent relative to the lipid layer whether inside or in the coating of the microparticles, thus providing information about the structure of the microparticles. The 1 mg/mL Fluorescein aqueous solution was introduced during the processing of the microparticles, more precisely, in the aqueous phase before the addition of the ethanolic solution of lipids or the Dexa or lipids (5% Disodium Fluoresceinum relative to the solid P). The resulting suspensions were kept at 4 °C in the dark.

#### 4.2.4. Suspensions of Lmp and Lmp/Dexa in PBS and Physical Mixture of Pure Dexa with Lmp

Preliminary tests on the behavior of pure Dexa, Lmp, and Lmp/Dexa in PBS were performed in order to study of the release of Dexa from Lmp/Dexa in PBS. In this regard, samples of pure Dexa, Lmp, and Lmp/Dexa were dispersed in PBS. The samples of Dexa–PBS, Lmp–PBS, and Lmp/Dexa–PBS recovered from the PBS were analyzed by FTIR and XRD and compared to pure Dexa, Lmp and Lmp/Dexa. In addition, a physical mixture of pure Dexa with Lmp in the ratio corresponding to the content of Dexa in Lmp/Dexa was prepared and analyzed in comparison to Lmp/Dexa.

#### 4.2.5. Encapsulation Efficiency of Dexa and Dexa Loading Efficiency in Lmp/Dexa

In order to calculate the encapsulation efficiency (E_ef_), the UV-Vis spectrophotometry quantitative method (SPECORD 250 PLUS spectrophotometer equipped with WinAspect software) was used. Dexa concentration was determined at λ = 243 nm based on the calibration curve, registered using five diluted solutions of pure Dexa in 50% (*v/v*) ethanol solution with concentrations between 0.001 mg/mL and 0.02 mg/mL.

After 24 h at 4 °C, the suspension containing Lmp/Dexa separated into two layers, a clear supernatant solution and a layer of white sediment. After removing the supernatant, both layers were measured considering the net volume of each fraction. The measured supernatant was used to evaluate the free Dexa concentration and to calculate E_ef_ (%). The supernatant was formed from the excess aqueous solution of P, to which was added the excess ethanolic solution containing dissolved free Dexa. Considering that Dexa is not water soluble, if the clear supernatant solution contains Dexa molecules they were certainly from the ethanolic solution in the synthesis stage. The measured volume of sediment was employed in the following in vitro studies. The sedimented Lmp/Dexa was washed with a small amount of distilled water and recovered. Using the Dexa calibration curve, the Dexa concentration in the removed supernatant solution containing Dexa remaining from the Dexa loading process (free Dexa) was determined. The E_ef_ for Dexa was estimated according to Equation (1) [51]:

E_ef_ (%) = (Total feeding Dexa − Free Dexa) × 100/Total feeding Dexa (1)

The Dexa loading Efficiency in Lmp/Dexa (Dexa_loadef_ (%)) was calculated according to Equation (2):

Dexa_loadef_ (%) = Total loaded Dexa × 100/Total Lmp (2)

where

Total loaded Dexa = Total feeding Dexa − Free Dexa(3)

#### 4.2.6. Methods for Structural Characterization of Lmp and Lmp/Dexa

##### FTIR Spectroscopy

FTIR spectroscopy was used to identify the functional groups of the Lmp and Lmp/Dexa by FT-IR spectra recorded using a JASCO-FTIR 610 spectrometer (Jasco Europe s.r.l. Cremella, Italy) equipped with an ATR (attenuated total reflectance) accessory with a horizontal ZnSe crystal (Jasco PRO400S). The spectra resolution was 4 cm^−1^, and scans were repeated 100 times. An appropriate amount of the samples was placed on the ZnSe crystal, then the FTIR spectrum was recorded in the range 4000–500 cm^−1^.

##### X-ray Diffraction

The influence of the entrapment process on the crystalline structure of Dexa was studied using X-ray diffraction (XRD). The diffraction patterns of the precursors, Lmp and Lmp/Dexa, were registered by an INEL EQUINOX 3000 diffractometer with CoKα radiation (λ = 1.7903Å) and a 2-theta angular range of 20–110°.

##### Thermal Analysis

The thermal stability of Lmp/Dexa compared with pure Dexa, Lmp, and Lmp + Dexa (the physical mixture) samples was investigated by Differential Scanning Calorimetry (DSC) and Thermogravimetric (TG) analyses using a DSC-TG Labsys Setaram Instrument. The samples were analyzed at a heat flow rate of 10 °C/min under Argon purging within the temperature range of 25 °C–350 °C using an aluminum crucible. A high purity alumina powder was used as a reference in the DSC measurements. DSC and TG measurements were carried out to establish possible interaction between the biopolymers and lipid components and to evaluate the stability of the Dexa encapsulation into the carriers. All experiments were run in triplicate.

##### Optical Microscopy

Optical microscopy can be a rapid, comfortable, brief, and non-resource intensive technique, and accordingly was used to follow up formation of the microparticles in terms of their shape and size and to control their stability over time. Samples of Lmp and Lmp/Dexa suspensions were deposited drop-wise onto cover glass and dried at room temperature. Images were captured using an Optika B-383FL microscope.

In order to highlight the parts of the Lmp system, we used an optical microscope equipped with a fluorescence illuminator. For this purpose, both samples, the one with intrinsic fluorescence due to the presence of BSA and the samples processed with disodium fluoresceinum, were used. The B filter (excitation at 475 nm and emission at 515 nm) and G filter (excitation at 530 nm and emission at 590 nm) f were used or fluorescence reflected light observation and compared with Bright field (BF) empty for transmitted light observation.

##### Scanning Electron Microscopy (SEM)

Although SEM is not usually used to characterize the morphology of liposomes because they do not withstand working conditions, degrading even more, obtaining relevant images of the proposed hybrid system confirms the stability of its structure. SEM micrographs were used to highlight the changes in the surface morphology of Lmp after Dexa loading. In this respect, samples of both Lmp and Lmp/Dexa suspensions were deposited drop-wise, without spreading the layer evenly, onto a cover glass and dried at room temperature. The dried samples were mounted on carbon sticky tabs and then covered with a 10 nm Au layer. Samples were analyzed using a Hitachi SU8230 scanning electron microscope at 30 kV, 10 uA and 8 mm working distance.

#### 4.2.7. Methods for the Study of Lmp/Dexa Characteristics

##### In Vitro Release Study

The in vitro release of Dexa from Lmp/Dexa was conducted into both water and PBS pH 7.4, each containing a small amount of ethanol (1%) to allow for Dexa solubility. The study of release from pure Dexa solution of the same Dexa concentration as in the microparticles was performed in 1% ethanol aqueous solution. The assessment of Dexa concentration in the release medium using UV-Vis Quant mode based on the calibration curve was performed. The Dexa release profile from Lmp/Dexa was developed using a previously reported dialysis method [52]. The study was conducted at 37 °C, under slow magnetic stirring at 100 rpm. A 1mL Lmp/Dexa sample and 1 mL 1% Ethanol solution/PBS pH 7.4 containing 1% ethanol were introduced in a dialysis bag (MWCO:3 kDa) and the sealed bag was immersed in 19 mL 1% Ethanol solution/PBS ph 7.4 containing 1% ethanol, the release medium, being placed into a 100 mL Erlenmeyer flask with a flat bottom and closed with ground glass stopper and left for 144 h. At certain time intervals, three samples of 1 mL release medium each were removed and replaced with the same volume of fresh medium. The sample was further incubated. The collected samples were diluted with 50% ethanol solution (*v*/*v*), homogenized, and then introduced into the spectrometer measuring cuvette for Dexa quantification at λ = 243 nm against the adequate calibration curve previously registered. All studies were performed in triplicate. The Dexa concentrations at any specified time t_n (1–32)_ (t_1_ = 5 min, t_2_ = 10 min, t_3_ = 15 min, t_4_ = 30 min, t_5_ = 45 min and t_6_ = 1 h, t_7_ = 2 h, t_8_ = 3 h, t_9_ = 4 h, t_10_ = 5 h, t_11_ = 6 h, t_12_ = 12 h, t_13_ = 24 h, t_14_ = 30 h, t_15_ = 36 h, t_16_ = 42 h, t_17_ = 48 h, t_18_ = 54 h, t_19_ = 60 h, t_20_ = 66 h, t_21_ = 72 h, t_22_ = 78 h, t_23_ = 84 h, t_24_ = 90 h, t_25_ = 102 h, t_26_ = 108 h, t_27_ = 114 h, t_28_ = 120 h, t_29_ = 126 h, t_30_ = 132 h, t_31_ = 138 h and t_32_ = 144 h) (C_n_) were determined.

The cumulative Dexa percent was calculated as the percent of released Dexa at a specific time n against the amount of Dexa estimated in the sample subjected to release, based on Dexa Loading_ef_ (%).

The cumulative release (%) of Dexa from the total Dexa before releasing was further plotted against the release time.

##### Cytotoxicity Assay of Lmp and Lmp/Dexa

1. Cell Lines, Cultures, and Treatments

For the in vitro study, we used the House Ear Institute-Organ of Corti 1 (HEI-OC1) cell line, derived from the auditory organ of a transgenic mouse “Immortomouse” [53]. The cells were cultured in Dulbecco’s Modified Eagles’ Medium (DMEM) supplemented with 10% fetal bovine serum (FBS) in permissive conditions of 33 °C and 10% CO_2_. These conditions allowed for the expression of an immortalizing gene that triggers de-differentiation and accelerated proliferation [54]. The HEI-OC1 cell line was a gift from Professor Federico Kalinek of the House Ear Institute (Los Angeles, CA, USA). This cell line is considered adequate for investigating the cellular and molecular mechanisms involved in ototoxicity and for screening of the potential ototoxicity or otoprotective properties of new pharmacological drugs [55].

Another cell line used in our experiments was the spontaneously immortalized human keratinocyte cell line HaCaT, purchased from the Cell Line Service of the German Cancer Research Centre in Heidelberg. The keratinocytes were cultured in high-glucose DMEM supplemented with 10% d FBS, 2 mM glutamine, 50 UI/mL penicillin, and 50 mg/mL streptomycin. The reason we used the HaCaT cell line was to compare the sensitivity towards the tested particles, having in mind that the HEI-OC1 cell line is derived from mouse cells and the HaCat cell line represents a human cellular line. We wanted to test the toxicity of the Lmp and Lmp/Dexa on a human cellular line, because the microcarriers have the end goal of treating acquired SNHL in human patients.as.

All media and substances were purchased from Sigma-Aldrich Chemie GmbH, Taufkirchen, Germany.

For the cytotoxicity experiments, the cells were plated in 96-well plates, (Nunc, Thermo Scientific, Waltham, MA, USA) 2 × 10^4^ cells/well. Treatments were carried out with serial dilutions of Lmp and Lmp/Dexa. For HEI-OC1 cells, the following concentrations were used: 50, 40, 20, 10, 5, 2, 1, and 0.1 μg/mL. The concentrations are expressed as the concentration of Dexamethasone contained in the microcapsules. For HaCaT cells, the concentrations were 100, 80, 50, 40, 20, 15, 7.5, and 3.75 μg/mL. For each experiment, cells in a minimum of three wells were left untreated as a control.

For the protection experiments on HEI-OC1 cells, 100 µM Cisplatin was added to each well 30 min after treatment with Lmp and Lmp/Dexa, a concentration close to the IC50 of Cisplatin. In addition, for comparison of the protection offered by Lmp/Dexa with a well-known protective agent, two concentrations of Dexamethasone (25 and 50 µg/mL) were used.

2. In Vitro Release Study

The viability of the cells after exposure to Lmp and Lmp/Dexa was assessed by the alamar Blue fluorimetric test from Molecular Probes (Invitrogen, Carlsbad, CA, USA). At 24 h after the treatments, 20 μL of alamarBlue reagent was added to each well and incubated in standard cell culture conditions for 1 h. The emitted fluorescence was read on a BioTek Synergy 2 microplate reader at 570 nm excitation and 585 nm emission. At each concentration, the surviving fraction was calculated as the fluorescence of the sample/fluorescence of control non treated cells × 100. Each experiment was repeated three times.

Another viability test used was the MTT (3-(4,5-dimethylthiazol-2-yl)-2,5-diphenyl-2H-tetrazolium bromide) test. The absorbance was read with a Tecan Sunrise microplate reader at 570 nm.

#### 4.2.8. Statistical Analysis

The results were analyzed using GraphPad Prism 5 Software. (San Diego, CA, USA) and Microsoft Excel. One-way ANOVA analysis of variance and Dunnett’s test were used to compare the means of different groups to the control (non-treated) group. A significant effect was considered for p values below 0.5.

## Figures and Tables

**Table 1:** Values of Dexa Encapsulation efficiency (%) and Dexa Loading efficiency for different ratios of Dexa:Lipoid S100:DDAB used in Lmp/Dexa preparation.

Dexa:Lipoid S100:DDAB Ratio	Dexa Encapsulation Efficiency (%) ± SD	Dexa Loading Efficiency (%) ± SD
2:10:1	83.07 ± 1.35	0.22 ± 0.007
3:10:1	89.86 ± 0.42	0.36 ± 0.009
4:10:1	92.5 ± 0.61	0.50 ± 0.01

## Data Availability

Not applicable.

## References

[1] World Health Organization (2021). World Report on Hearing. https://www.who.int/publications/i/item/world-report-on-hearing.

[2] Wilson W.R., Byl F.M., Laird N. (1980). The Efficacy of Steroids in the Treatment of Idiopathic Sudden Hearing Loss: A Double-blind Clinical Study. Arch. Otolaryngol..

[3] Kuhn M., Heman-Ackah S.E., Shaikh J.A., Roehm P.C. (2011). Sudden Sensorineural Hearing Loss: A Review of Diagnosis, Treatment, and Prognosis. Trends. Amplif..

[4] Shemirani N.L., Schmidt M., Friedland D.R. (2009). Sudden sensorineural hearing loss: An evaluation of treatment and management approaches by referring physicians. Otolaryngol. Neck. Surg..

[5] Waissbluth S., Peleva E., Daniel S.J. (2017). Platinum-induced ototoxicity: A review of prevailing ototoxicity criteria. Eur. Arch. Oto-Rhino-Laryngol..

[6] Breglio A.M., Rusheen A.E., Shide E.D., Fernandez K.A., Spielbauer K.K., McLachlin K.M., Hall M.D., Amable L., Cunningham L.L. (2017). Cisplatin is retained in the cochlea indefinitely following chemotherapy. Nat. Commun..

[7] Yu D., Gu J., Chen Y., Kang W., Wang X., Wu H. (2020). Current Strategies to Combat Cisplatin-Induced Ototoxicity. Front. Pharmacol..

[8] Hill G.W., Morest D.K., Parham K. (2008). Cisplatin-Induced Ototoxicity: Effect of intratympanic dexamethoasone injections. Otol. Neurotol..

[9] Schreiber B.E., Agrup C., Haskard D.O., Luxon L.M. (2010). Sudden sensorineural hearing loss. Lancet.

[10] Nyberg S., Abbott N.J., Shi X., Steyger P.S., Dabdoub A. (2019). Delivery of therapeutics to the inner ear: The challenge of the blood-labyrinth barrier. Sci. Transl. Med..

[11] Williams D.M. (2018). Clinical pharmacology of corticosteroids. Respir. Care.

[12] Dindelegan M.G., Blebea C., Perde-Schrepler M., Buzoianu A.D., Maniu A.A. (2022). Recent Advances and Future Research Directions for Hearing Loss Treatment Based on Nanoparticles. J. Nanomater..

[13] Salt A.N., Plontke S.K. (2005). Local inner-ear drug delivery and pharmacokinetics. Drug Discov. Today.

[14] Dormer N.H., Nelson-Brantley J., Staecker H., Berkland C.J. (2019). Evaluation of a transtympanic delivery system in Mus musculus for extended release steroids. Eur. J. Pharm. Sci..

[15] Yang K.-J., Son J., Jung S.Y., Yi G., Yoo J., Kim D.-K., Koo H. (2018). Optimized phospholipid-based nanoparticles for inner ear drug delivery and therapy. Biomaterials.

[16] Egli Gallo D., Khojasteh E., Gloor M., Hegemann S.C. (2013). Effectiveness of systemic high-dose dexamethasone therapy for idiopathic sudden sensorineural hearing loss. Audiol. Neurotol..

[17] Li X., Chen W.-J., Xu J., Yi H.-J., Ye J.-Y. (2021). Clinical Analysis of Intratympanic Injection of Dexamethasone for Treating Sudden Deafness. Int. J. Gen. Med..

[18] Czock D., Keller F., Rasche F.M., Häussler U. (2005). Pharmacokinetics and Pharmacodynamics of Systemically Administered Glucocorticoids. Clin. Pharmacokinet..

[19] Liu D., Ahmet A., Ward L., Krishnamoorthy P., Mandelcorn E.D., Leigh R., Brown J.P., Cohen A., Kim H. (2013). A practical guide to the monitoring and management of the complications of systemic corticosteroid therapy. Allergy Asthma Clin. Immunol..

[20] Toniazzo T., Berbel I.F., Cho S., Fávaro-Trindade C.S., Moraes I.C., Pinho S.C. (2014). β-carotene-loaded liposome dispersions stabilized with xanthan and guar gums: Physico-chemical stability and feasibility of application in yogurt. LWT.

[21] Guldiken B., Gibis M., Boyacioglu D., Capanoglu E., Weiss J. (2018). Physical and chemical stability of anthocyanin-rich black carrot extract-loaded liposomes during storage. Food Res. Int..

[22] Lin W., Goldberg R., Klein J. (2022). Poly-phosphocholination of liposomes leads to highly-extended retention time in mice joints. J. Mater. Chem. B.

[23] Tan C., Wang J., Sun B. (2021). Biopolymer-liposome hybrid systems for controlled delivery of bioactive compounds: Recent advances. Biotechnol. Adv..

[24] Shah S., Famta P., Raghuvanshi R.S., Singh S.B., Srivastava S. (2021). Lipid polymer hybrid nanocarriers: Insights into synthesis aspects, characterization, release mechanisms, surface functionalization and potential implications. Colloids Interface Sci. Commun..

[25] Bhargavi N., Dhathathreyan A., Sreeram K. (2020). Regulating structural and mechanical properties of pectin reinforced liposomes at fluid/solid interface. Food Hydrocoll..

[26] Zhou W., Liu W., Zou L., Liu W., Liu C., Liang R., Chen J. (2014). Storage stability and skin permeation of vitamin C liposomes improved by pectin coating. Colloids Surfaces B Biointerfaces.

[27] Shao P., Wang P., Niu B., Kang J. (2018). Environmental stress stability of pectin-stabilized resveratrol liposomes with different degree of esterification. Int. J. Biol. Macromol..

[28] Prajapati V.D., Jani G.K., Moradiya N.G., Randeria N.P. (2013). Pharmaceutical applications of various natural gums, mucilages and their modified forms. Carbohydr. Polym..

[29] Rana V., Rai P., Tiwary A.K., Singh R.S., Kennedy J.F., Knill C.J. (2011). Modified gums: Approaches and applications in drug delivery. Carbohydr. Polym..

[30] Van Bracht E., Raavé R., Verdurmen W.P.R., Wismans R.G., Geutjes P.J., Brock R.E., Oosterwijk E., van Kuppevelt T.H., Daamen W.F. (2012). Lyophilisomes as a new generation of drug delivery capsules. Int. J. Pharm..

[31] Kratz F. (2008). Albumin as a drug carrier: Design of prodrugs, drug conjugates and nanoparticles. J. Control. Release.

[32] Grinberg O., Hayun M., Sredni B., Gedanken A. (2007). Characterization and activity of sonochemically-prepared BSA microspheres containing Taxol—An anticancer drug. Ultrason. Sonochemistry.

[33] Shen H.J., Shi H., Ma K., Xie M., Tang L.L., Shen S., Li B., Wang X.-S., Jin Y. (2013). Polyelectrolyte capsules packaging BSA gels for pH-controlled drug loading and release and their antitumor activity. Acta Biomater..

[34] Yu S., Hu J., Pan X., Yao P., Jiang M. (2006). Stable and pH-sensitive nanogels prepared by self-assembly of chitosan and ovalbumin. Langmuir.

[35] Lu B., Xiong S.-B., Yang H., Yin X.-D., Zhao R.-B. (2006). Mitoxantrone-loaded BSA nanospheres and chitosan nanospheres for local injection against breast cancer and its lymph node metastases: I: Formulation and in vitro characterization. Int. J. Pharm..

[36] Shah S., Dhawan V., Holm R., Nagarsenker M.S., Perrie Y. (2020). Liposomes: Advancements and innovation in the manufacturing process. Adv. Drug Deliv. Rev..

[37] Jaafar-Maalej C., Diab R., Andrieu V., Elaissari A., Fessi H. (2009). Ethanol injection method for hydrophilic and lipophilic drug-loaded liposome preparation. J. Liposome Res..

[38] Butler M.F., Glaser N., Weaver A.C., Kirkland A.M., Heppenstall-Butler M. (2006). Calcium Carbonate Crystallization in the Presence of Biopolymers. Cryst. Growth Des..

[39] Cipolla D., Wu H., Salentinig S., Boyd B., Rades T., Vanhecke D., Petri-Fink A., Rothin-Rutishauser B., Eastman S., Redelmeier T. (2016). Formation of drug nanocrystals under nanoconfinement afforded by liposomes. RSC Adv..

[40] Beck R., Pohlmann A., Hoffmeister C., Gallas M., Collnot E., Schaefer U., Guterres S., Lehr C.-M. (2007). Dexamethasone-loaded nanoparticle-coated microparticles: Correlation between in vitro drug release and drug transport across Caco-2 cell monolayers. Eur. J. Pharm. Biopharm..

[41] Gómez-Gaete C., Fattal E., Silva L., Besnard M., Tsapis N. (2008). Dexamethasone acetate encapsulation into Trojan particles. J. Control. Release.

[42] Dukovski B.J., Plantić I., Čunčić I., Krtalić I., Juretić M., Pepić I., Lovrić J., Hafner A. (2017). Lipid/alginate nanoparticle-loaded in situ gelling system tailored for dexamethasone nasal delivery. Int. J. Pharm..

[43] Bucatariu S., Constantin M., Ascenzi P., Fundueanu G. (2016). Poly(lactide-co-glycolide)/cyclodextrin (polyethyleneimine) microspheres for controlled delivery of dexamethasone. React. Funct. Polym..

[44] Hsiao I.L., Gramatke A.M., Joksimovic R., Sokołowski M., Gradzielski M., Haase A. (2014). Size and Cell Type Dependent Uptake of Silica Nanoparticles. J. Nanomed. Nanotechnol..

[45] Kroll A., Dierker C., Rommel C., Hahn D., Wohlleben W., Schulze-Isfort C., Göbbert C., Voetz M., Hardinghaus F., Schnekenburger J. (2011). Cytotoxicity screening of 23 engineered nanomaterials using a test matrix of ten cell lines and three different assays. Part. Fibre Toxicol..

[46] Rees K.R. (1980). Cells in Culture in Toxicity Testing: A Review. J. R. Soc. Med..

[47] Perde-Schrepler M., Fischer-Fodor E., Virag P., Brie I., Cenariu M., Pop C., Valcan A., Gurzau E., Maniu A. (2020). The expression of copper transporters associated with the ototoxicity induced by platinum-based chemotherapeutic agents. Hear. Res..

[48] Kalinec G., Thein P., Park C., Kalinec F. (2016). HEI-OC1 cells as a model for investigating drug cytotoxicity. Hear. Res..

[49] Pons M., Foradada M., Estelrich J. (1993). Liposomes obtained by the ethanol injection method. Int. J. Pharm..

[50] Charcosset C., Juban A., Valour J.-P., Urbaniak S., Fessi H. (2015). Preparation of liposomes at large scale using the ethanol injection method: Effect of scale-up and injection devices. Chem. Eng. Res. Des..

[51] Anitha A., Maya S., Deepa N., Chennazhi K., Nair S., Tamura H., Jayakumar R. (2011). Efficient water soluble O-carboxymethyl chitosan nanocarrier for the delivery of curcumin to cancer cells. Carbohydr. Polym..

[52] Paşcalău V., Tertis M., Pall E., Suciu M., Marinca T., Pustan M., Merie V., Rus I.A., Moldovan C., Topala T. (2020). Bovine serum albumin gel/polyelectrolyte complex of hyaluronic acid and chitosan based microcarriers for Sorafenib targeted delivery. J. Appl. Polym. Sci..

[53] Kalinec G.M., Webster P., Lim D.J., Kalinec F. (2003). A Cochlear Cell Line as an in vitro System for Drug Ototoxicity Screening. Audiol. Neurotol..

[54] Devarajan P., Savoca M., Castaneda M., Park M.S., Esteban-Cruciani N., Kalinec G., Kalinec F. (2002). Cisplatin-induced apoptosis in auditory cells: Role of death receptor and mitochondrial pathways. Hear. Res..

[55] Kalinec G.M., Park C., Thein P., Kalinec F. (2016). Working with Auditory HEI-OC1 Cells. J. Vis. Exp..

